# Overlapping Localization of MAP65-2, -6, and -7 in Arabidopsis Hypocotyl Cells.

**DOI:** 10.17912/micropub.biology.000971

**Published:** 2023-10-24

**Authors:** Jessica R Lucas, Sidney L Shaw

**Affiliations:** 1 Biology, University of Wisconsin - Oshkosh; 2 Biology, Indiana University, Bloomington, Indiana, United States

## Abstract

Microtubules are essential components of eukaryotic cells. Myriad proteins associate with microtubules to facilitate the organization and operation of microtubule arrays. Various M icrotubule A ssociated P roteins (MAPs) assist the assembly and function of mitotic spindles and interphase arrays. Nine MAP65 genes exist in the genome of the acentrosomal model plant, *Arabidopsis thaliana,* and the function of majority of these proteins is unclear. To address this knowledge gap, we demonstrate the localization of *A. thaliana* MAP65-6 and MAP65-7 fusion proteins expressed from native promoters in interphase cells of developing *A. thaliana* seedlings. Analyses of these fusion proteins co-expressed with alpha-tubulin 6 reporters indicate that MAP65-6 and MAP65-7 bind a subset of interphase microtubules. Co-expression of GFP: MAP65-6 with mCherry: MAP65-2 from native promoters in *A. thaliana* showed overlapping localization patterns on interphase microtubule bundles. Collectively, these data suggested that MAP65-2 , -6, and -7 bind cortical microtubule bundles in plant interphase microtubule arrays.

**Figure 1. MAP65-6 and MAP65-7 expression in interphase epidermal cells of developing A. thaliana seedlings.**
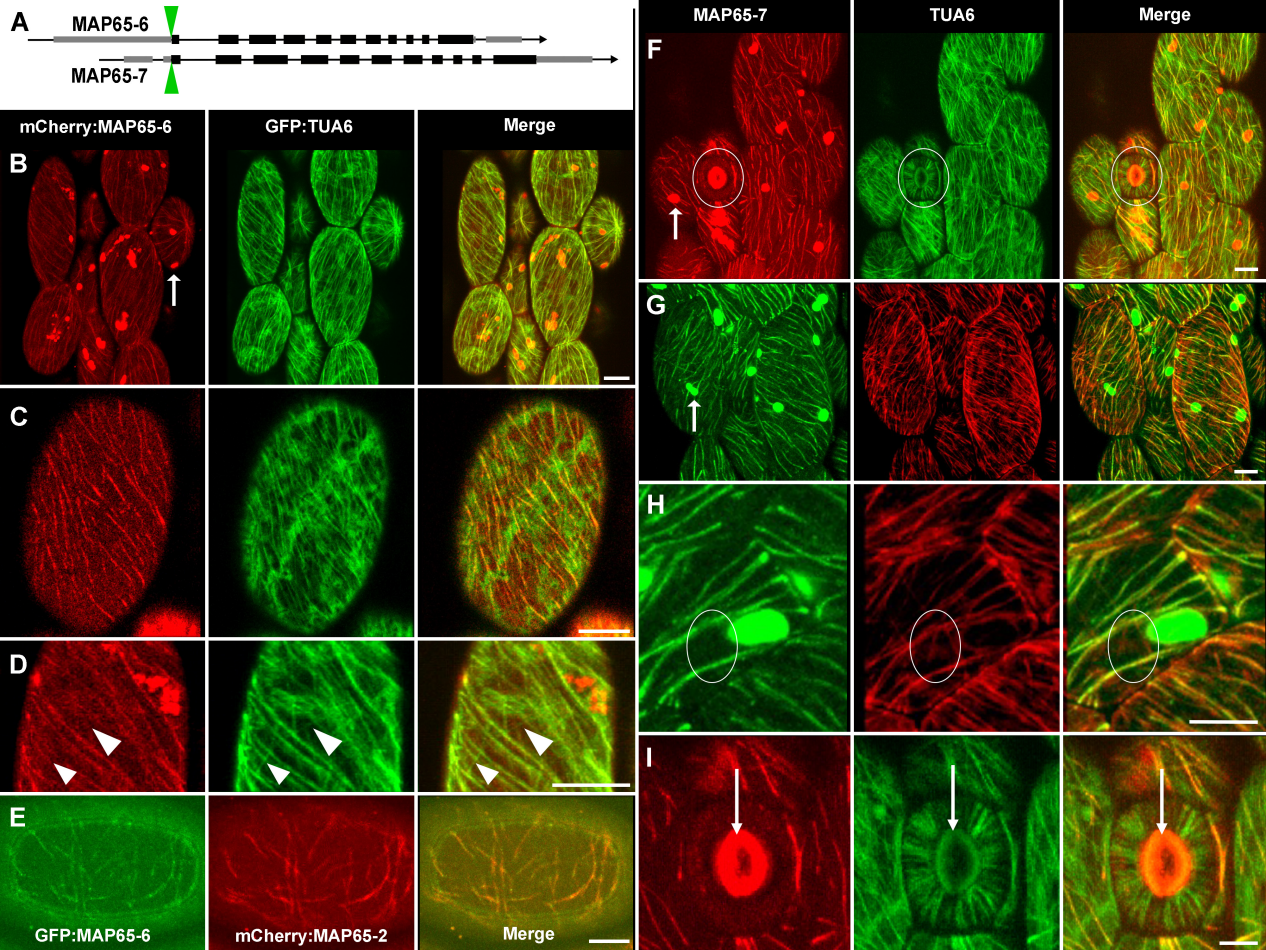
A) Schematic representation of *A. thaliana* MAP65-6 and MAP65-7 genes showing N-terminal position of fluorescent proteins and illustrating similarity of MAP65-6 and -7 gene structure. Exons represented as black rectangles, introns as black lines between exons, and untranslated regions as grey rectangles. All confocal images shown in this panel were *A. thaliana* interphase hypocotyl cells. B-D) Co-expression of mCherry: MAP65-6 driven from native promoter (red, left panels) with constitutively expressed 35S:GFP:alpha tubulin6 (green, central panels). Merged images of mCherry:MAP65-6 and GFP:TUA6 on right. B) mCherry:MAP65-6 labeled microtubules, and arrow in left panel marks a representative non-microtubule structure labeled with mCherry:MAP65-6. C) mCherry:MAP65-6 localized to a subset of interphase microtubules. Note that GFP:TUA6 integrated into linear cortical microtubules and accumulated in the underlying cortical cytoplasm (center). D) Magnified view of mCherry:MAP65-6 and GFP:TUA6. Arrowheads indicate single GFP:TUA6 labeled microtubules (green lines in center and right panels) that were not labelled with mCherry:MAP65-6 (left). E) Overlapping localization of GFP:MAP65-6 and mCherry: MAP65-2 , both from native promoters. F-I) Co-expression of native promoter MAP65-7 and TUA6 transgenes. Left panels demonstrated native promoter:mCherry:MAP65-7 (F and I) and native promoter:GFP:MAP65-7 (G and H). Microtubules shown as GFP:TUA6 (center panels F and I) or mCherry:TUA6 (G and H). Green and red channels were merged in right panels. F) MAP65-7 labeled interphase microtubules in all epidermal cells. White circular outline in F marked stomatal guard cells, which are shown in higher magnification in I. Arrows in F and G indicated non-microtubule structures labeled with MAP65-7. G) Native promoter:GFP:MAP65-7 and mCherry:TUA6 co-expression reiterated that MAP65-7 labeled a subpopulation of interphase cortical microtubules and cytoplasmic compartments. H) White ovals surrounded two microtubules (faint red lines in center and right panels) not labeled with GFP:MAP65-7 (left panel). I) Stomatal guard cells labeled with mCherry:MAP65-7 (left) and GFP:TUA6 (center); downward arrow pointed to stomatal pore which auto-fluoresced in red and green channels. MAP65-7 sparsely labeled few cortical microtubules in guard cells and epidermal cells. All mag bars = 10um.

## Description

M icrotubule A ssociated P roteins (MAPs) assist microtubule array organization and function in numerous ways throughout the eukaryotic cell cycle. In addition to the mitotic spindle, plant cells organize the preprophase band and phragmoplast microtubule arrays that predict and execute cytokinesis, respectively (Lucas *et al.* 2006). The plant interphase microtubule cytoskeleton transitions between multiple organizations in response to developmental cues, physiological states, and environmental stimuli (Vineyard *et al.* 2013, Lucas and Shaw 2008). Multiple MAPs facilitate array organization and MAPs mediate the role of interphase microtubules in growth and morphogenesis through cell wall formation, differential growth, and coordination with the actin cytoskeleton (Hamada 2014, Lucas and Shaw 2008) . Despite the widespread importance of MAPs in biology, many plant MAPs remain unstudied and uncharacterized in cells (Nebenfuhr and Dixit 2018, Struk and Dhonukshe 2014).

The cellular roles of *Arabidopsis thaliana* MAP65-6 and MAP65-7 proteins are unknown. Specific MAP65 family members are known to facilitate mitosis by bundling antiparallel microtubules in the central mitotic spindle (She *et al.* 2019, Walczak and Shaw 2010, Sasabe and Machida 2006). Nine MAP65 genes (designated MAP65-1 through MAP65-9 ) exist in the model plant *A. thaliana* and are split into five sub-paralogous groups (Smertenko *et al.* 2008). The role of *A. thaliana* MAP65-3 and 65-4 appear similar to canonical animal MAP65 proteins in antiparallel microtubule bundling during cell division (Li *et al.* 2017, Ho *et al.* 2012, Fache *et al.* 2010, Caillaud *et al.* 2008, Muller *et al.* 2004). MAP65-1 and MAP65-2 act during axial cell elongation and cell proliferation (Lucas and Shaw 2012, Lucas *et al.* 2011, Sasabe *et al.* 2011) and may provide microtubule polymers protection from severing, depolymerization from salt and/or low temperatures (Burkart and Dixit 2019, Zhou *et al* . 2017, Meng *et al.* 2010).

Previous studies indicated that *A. thaliana* MAP65 family members exhibited different biochemical properties, subcellular localizations, and expression patterns within plants (Stoppin-Mellet *et al.* 2013, Smertenko *et al.* 2008, Van Damme *et al.* 2004). Published reports of MAP65 subcellular localization are inconsistent which may be due to differences between immunolocalization and fluorescent reporter studies using heterologous and/or constitutive overexpression of fluorescent protein fusions in plants and cultured cells (Boruc *et al.* 2017, Meng *et al.* 2010, Smertenko *et al.* 2008, Damme *et al.* 2004). For example, *in vitro* studies suggested *A. thaliana* MAP65-6 crosslinks microtubules into a perpendicular meshwork while MAP65-1 bundles microtubules into linear arrays (Mao *et al.* 2005). Immunolocalization of MAP65-6 , however, indicated binding to linear microtubule arrays in the plant-specific preprophase band and phragmoplast (Smertenko *et al.* 2008). A separate antibody study indicated that MAP65-6 labeled mitochondria but not microtubules (Mao *et al.* 2005). The localization of MAP65-7 has not yet been published.

Here we report the localization of MAP65-6 and MAP65-7 fluorescent reporter proteins expressed from native genomic promoters on interphase cortical microtubules in *A. thaliana* seedlings. MAP65-6 (At2g019010) and MAP65-7 (At1g14690) proteins are 79.8% identical at the amino acid level. We built N-terminal eGFP and mCherry fusions to both *A. thaliana* MAP65-6 and MAP65-7 genomic sequences (Fig 1A), as a C-terminal fusion may interfere with protein function (Smertenko *et al,* 2008). Native promoters were used to examine where expression occurred in the plant and because overexpression of microtubule associated proteins may disrupt protein localization patterns (Zhang *et al.* 2020).

MAP65-6 and MAP65-7 were both expressed in the majority of light-grown *A. thaliana* epidermal hypocotyl cells and both labeled a subset of interphase cortical microtubules (Figures 1B and 1F). While both MAP65-6 and MAP65-7 fusion proteins bound apparent microtubule bundles within the cortical arrays, not all GFP:TUA6 or mCherry:TUA6 microtubule structures were MAP65 labeled (Figures 1C, D, G, H). Apparently single cortical microtubules were not labeled with MAP65-6 (arrowheads in Figure 1D ) or MAP65-7 (white oval in Figure 1H ). Microtubules in stomatal guard cells were sparsely labeled with mCherry:MAP65-7 (Figures 1F and I). The scant MAP65-7 labeling in guard cells could have resulted from weak expression of the native promoter:mCherry:MAP65-7 transgene and/or the predominance of parallel microtubule bundles in guard cells (Dixit *et al.* 2006). mCherry: MAP65-6 , mCherry: MAP65-7 , and GFP:MAP65-7 fusion proteins localized to non-microtubule cytoplasmic structures (arrows in Figure 1B, 1F, and 1G).

To better define the cortical microtubule subpopulation bound by MAP65-6 and MAP65-7 , we crossed *A. thaliana* plants expressing GFP: MAP65-6 and mCherry: MAP65-2 fusion proteins both expressed from native promoters. *A. thaliana* mCherry: MAP65-2 labeled microtubule bundles (Lucas *et al.* 2011, Lucas and Shaw 2012), and co-localized with GFP: MAP65-6 in seedling stems ( Figure 1E ). Within the *A. thaliana* MAP65 family, MAP65-2 (At4g26760) shares 45.6% and 46.1% amino acid identity with MAP65-6 and MAP65-7 respectively.

In conclusion, *A. thaliana* MAP65-6 and MAP65-7 fluorescent reporter protein fusions driven from native promoters in living cells decorated a subset of interphase microtubules ( Figure 1 ) and replicate prior findings for MAP65-1 and MAP65-2 (Lucas et al., 2011) . These data contrast somewhat with a previous MAP65-6 immunofluorescence study of cell division related microtubule arrays (Smertenko *et al.* 2008), possibly due to cell type and/or cell cycle specific regulation. Further studies will determine the identity of the intracellular compartments labeled with these MAP65-6 and MAP65-7 fusion proteins. Co-expression of these reporters with tubulin or MAP65-2 reporters indicated that MAP65-6 decorated antiparallel bundles. Furthermore, parallel microtubule bundles in stomatal guard cells were not labeled with mCherry: MAP65-7 . As MAP65-6 and MAP65-7 are nearly 80% similar to one another and show similar subcellular localizations, their functional roles may overlap. Further genetic analysis will be needed to determine protein function and assess potential redundancy with other MAP65 proteins in plant cells.

## Methods

*Construction of Arabidopsis thaliana plants expressing fusion proteins.*

MAP65-6 (At2g01910) and MAP65-7 (At1g14690) full genome sequences were cloned from wild type Columbia-0 *A. thaliana* genomic DNA into p211 plasmid so the GFP coding sequence was N-terminal to the MAP65 coding sequence. The native promoter (2.5kB of upstream genomic DNA sequence upstream) of both MAP65-6 and 65-7 were cloned to drive the expression of the respective N-terminal GFP fusion. Each construct was transformed by *Agrobacterium* floral dip transformation into homozygous T-DNA SALK lines for the respective gene ( MAP65-6 , SALK_020795; MAP65-7 , SALK_079592). Detailed protocols on plasmid and transgene construction, transformation, and selection, see Lucas *et al* 2011.

Transgenic lines were produced by dipping developing floral stems of *A. thaliana* plants into liquid cultures of *Agrobacterium tumenfaciens* harboring Ti plasmids with engineered transgenes (Clough and Bent 1998) . Transgenic lines expressing both MAP65 and alpha tubulin 6 reporters were built by dipping floral stems into *A. tumenfaciens* cultures containing both transgenes. Mature seeds from dipped plants dried for two weeks and were then sown on 1/2x Murishage and Skoog nutrient agar plates agar plates supplemented with kanamycin and/or hygromycin antibiotics. Seeds were germinated in a Percival growth chamber for 12-14 days to select for genetic transformation. T1 seedlings resistant to antibiotics were transplanted to soil, grown to maturity, and produced progeny T2 seeds through self-fertilization. These T2 seeds were then germinated in 24 hours of light on nutrient agar plates, and 6-day old hypocotyls were imaged on a Leica SP5 confocal microscope. At least six T2 seedlings from five different T1 parent plants were viewed for each of four transformations (GFP: MAP65-6 with mCherry:TUA6, GFP: MAP65-7 with mCherry:TUA6, mCherry: MAP65-6 with mGFP:TUA6, and mCherry:MAP65-7 with GFP:TUA6). All seedlings showed similar microtubule localizations of MAP65-6 and MAP65-7 on interphase cortical microtubules.

*Live-cell Confocal Imaging of Reporter Gene Fusions*

Seedlings were imaged on a Leica SP5 microscope with 488-nm and 561-nm laser excitation lines to excite eGFP and mCherry reporter fusions, respectively. Cells were viewed with 63x water immersion lens (1.2 N.A.). Seedlings were mounted on glass slides in liquid nutrient media under 1.5 coverslips. Vacuum grease adhered the coverslip to the slide and protected the seedling from compression. Plants acclimated to slides for 20 minutes before imaging.
